# Cementless Oxford Medial Unicompartmental Knee Replacement—Clinical and Radiological Results of 228 Knees with a Minimum 2-Year Follow-Up

**DOI:** 10.3390/jcm9051476

**Published:** 2020-05-14

**Authors:** Benjamin Panzram, Mira Mandery, Tobias Reiner, Tobias Gotterbarm, Marcus Schiltenwolf, Christian Merle

**Affiliations:** Department of Orthopaedic and Trauma Surgery, University Hospital Heidelberg, Schlierbacher Landstrasse 200a, 69118 Heidelberg, Germany; mira.mandery@gmail.com (M.M.); Tobias.Reiner@med.uni-heidelberg.de (T.R.); Tobias.Gotterbarm@kepleruniklinikum.at (T.G.); Marcus.Schiltenwolf@med.uni-heidelberg.de (M.S.); Christian.Merle@med.uni-heidelberg.de (C.M.)

**Keywords:** cementless UKR, OUKR, radiolucent lines, radiolucencies, cementless fixation, Oxford medial

## Abstract

(1) Background: Studies show several advantages of unicompartmental knee replacement (UKR) over total knee replacements (TKR), whereas registry based revision rates of UKR are significantly higher than for TKA. Registry data report lower revision rates for cementless UKR compared to cemented UKR. The aim of this study was to assess clinical and radiological results of cementless Oxford UKR (OUKR) in an independent cohort. (2) Methods: This retrospective cohort study examines a consecutive series of 228 cementless OUKR. Clinical outcome was measured using functional scores (Oxford Knee Score (OKS), American Knee Society Score (AKSS), Hannover Functional Ability Questionnaire for Osteoarthritis (FFbH-OA), range of motion (ROM)), pain and satisfaction. Radiographs were analyzed regarding the incidence of radiolucent lines (RL), implant positioning, and their possible impact on clinical outcome. (3) Results: At a mean follow-up of 37.1 months, the two and three year revision free survival-rates were 97.5% and 96.9%. Reasons for revision surgery were progression of osteoarthritis, inlay dislocation and pain. All clinical outcome scores showed a significant improvement from pre- to postoperative. The incidence of RL around the implant was highest within the first year postoperatively (36%), and decreased (5%) within the second year. Their presence was not correlated with inferior clinical outcome. Implant positioning showed no influence on clinical outcome. (4) Conclusion: Cementless OUKR showed excellent clinical outcome and survival rates, with reliable osteointegration. Neither the incidence of radiolucent lines nor implant positioning were associated with inferior clinical outcome.

## 1. Introduction

Unicompartmental knee replacement (UKR) is considered as a viable treatment option for patients with unicompartmental knee osteoarthritis (OA). Expert centers have demonstrated excellent functional outcome and high survival rates. However, higher revision rates compared to total knee replacement (TKR) have been reported by various registries, even though functional outcome is better, recovery time is faster, and cost-efficiency is higher [1,2,3,4].

Most available unicompartmental knee replacements rely on bone cement for the fixation of the implant within the bone. The cementless alternative of the Oxford UKR is coated with porous titanium and hydroxyapatite to facilitate osteointegration of the implant. Possible advantages of the cementless version are the reduced duration of surgery, the absence of cement-associated complications, such as cementation errors or the development of loose cement bodies within the joint, which may lead to revision surgery.

Recent registry data show lower revision rates for cementless than for cemented UKR [5]. A possible explanation for this may be the lower incidence of radiolucent lines (RL) in cementless UKR. If falsely interpreted as signs of loosening, their occurrence may lead to unnecessary revision [6,7,8].

While a few studies demonstrate excellent clinical outcome for cementless UKR [6,9,10,11,12], there is a lack of independent studies investigating the clinical impact of radiolucent lines (RL), that occur early and particularly at the tibial bone–implant interface.

With this study, we aimed to assess clinical and radiological outcome of cementless OUKR and to evaluate the clinical impact of RL and implant positioning.

## 2. Materials and Methods

This single-center cohort study included 228 consecutively implanted OUKR in 211 patients at our institution, between October 2012 and October 2015, with a minimum follow-up of two years. Surgery was indicated using Oxford criteria as previously described [13,14]. In all cases, patients suffered from anteromedial osteoarthritis (AMOA), and varus deformity was fully manually correctable. Obesity, age and cartilage loss in the patellofemoral joint were not considered as contraindications, as well as previous anterior cruciate ligament (ACL) reconstruction. The lateral compartment on valgus stress x-ray images was fully preserved [15]. Operations were performed by senior surgeons, being experienced with the OUKR system.

The institutional review board approved all procedures (S-326/2017). All patients gave written informed consent prior to inclusion. The study was conducted in accordance with the Helsinki Declaration as revised in 2013.

Clinical outcome was measured using the Oxford Knee Score (OKS, from 0 to 48 points), the American Knee Society Score (Objective: AKSS-O, Functioning: AKSS-F, from 0 to 100 points), and the Hannover Functional Ability Questionnaire for Osteoarthritis (FFbH-OA from 0% to 100%) [16].

Pain was measured on a visual analogue scale (VAS from 0 to 10) as well as overall satisfaction with the operation (VAS from 1—“extremely satisfactory”—to 5—“unsatisfactory”). Data acquisition was performed either by clinical follow-up exams (159 knees in 146 patients) or, if patients were unable to attend an exam, by questionnaires gathered by mail (33 knees in 31 patients).

**Figure 1:**
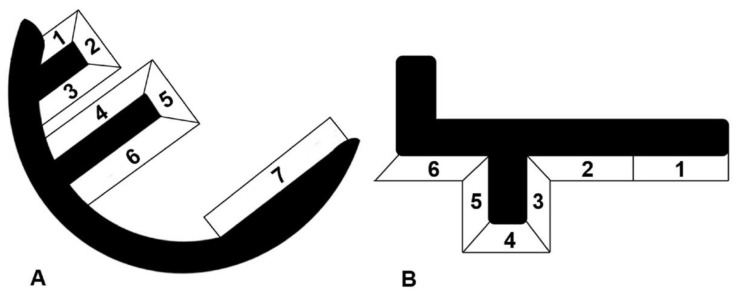
Zones of radiolucent lines (**A**)—femoral, (**B**)—tibial.

All radiographs were taken under fluoroscopical guidance as described by Gulati et al. [7]. For all knees included in the radiological evaluation, screened radiographs were taken postoperatively, and after a minimum follow-up of two years. The weight-bearing six tibial and seven femoral zones were inspected for the incidence of radiolucencies, as well as their development over time (Figure 1) [7,8,9].

Additionally, radiographs were examined for implant positioning. Four angles were measured: the varus–valgus angle of both components, as well as the tibial slope and extension–flexion angle of the femoral component, according to the manufacturer’s instructions [15,16,17].

**Figure 2:**
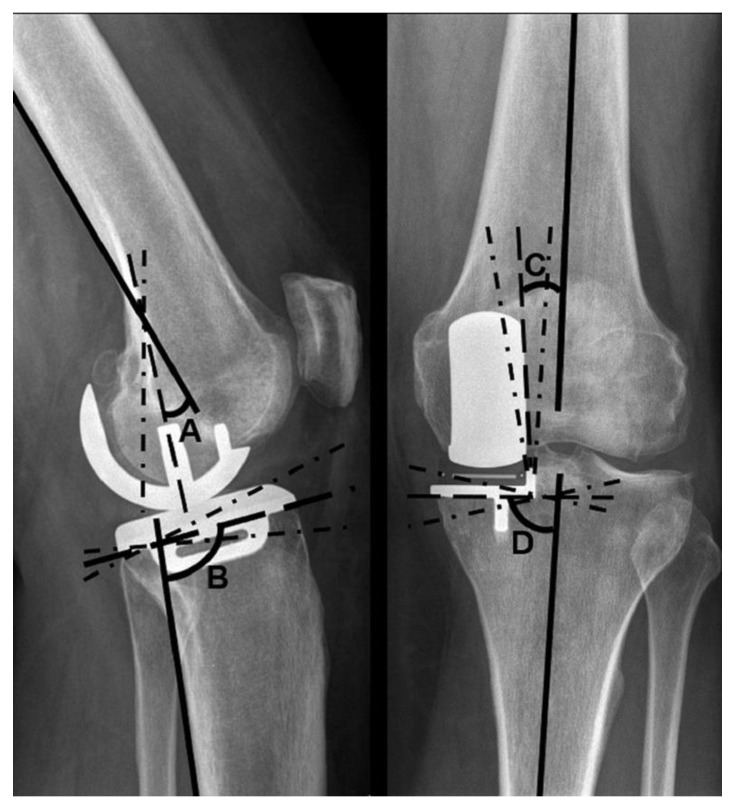
Implant positioning: **A**—femoral flexion (15° flexion, 0° extension tolerated), **B**—tibial slope: 97° standard value (±5° tolerated) **C**—femoral varus–valgus: 0° standard value (±10° tolerated) **D**—tibial varus–valgus: 90° standard value (±5° tolerated).

The varus–valgus angles of the components are stated as deviations from the anatomical axis of the distal femur and the proximal tibia, respectively. Varus deviations are described as positive, valgus deviations as negative values. The target range for femoral varus–valgus was ±10°, and for tibial varus–valgus ±5°. An increased tibial slope in relation to the reference value is stated as a positive degree value, a decreased tilt as a negative degree value. The target range for the tibial slope was 97° ± 5°. A flexion of the femoral component in relation to the femoral axis is described using positive degree values, a possible extension using negative degree values. A flexion of 15° and no extension were considered to be within the target range (Figure 2). 

Data analysis was conducted using IBM SPSS Statistics version 25.0 (IBM Corp., Armonk, N.Y., USA). 

Kaplan–Meier analysis was used for survival analysis, with revision surgery (surgery with removal/exchange of ≥1 part of the initially used implant) and re-operation (all other interventions under anesthesia) as endpoints. Patients were censored at death, revision surgery or last follow-up.

The clinical outcome scores were compared to their preoperative equivalent using Wilcoxon tests. Mann–Whitney U-testing was used to compare patients with and without RL, in terms of clinical and radiological outcome (OKS, AKSS-O, AKSS-F, FFbH-OA, range of motion (ROM), pain, satisfaction and implant positioning). The comparison between patients presenting implant angles within and outside the target zones was assessed respectively.

The incidence of RL in reference to outliers of implant positioning was evaluated using chi-square test. To counteract the problem of multiple testing, the *p*-value for statistical significance was set at *p* < 0.01.

## 3. Results

A total of 192 knees in 177 patients were included in the clinical, and 164 knees (152 patients) in the radiological follow-up. All patients had a minimum follow up of two years. In 90 knees, a follow-up of three years and longer was obtained. Mean follow-up was 37.1 months (SD: 9.8; range: 24–60; median: 35.0; interquartile range (IQR): 29–44).

The cohort included 95 male (53.7%) and 82 female (46.3%) patients, with a mean age at time of surgery of 61.3 years (SD: 9.7; range: 36–80) and at follow-up of 64.4 years (SD: 9.7; range: 38–82). The mean BMI at follow-up was 30.9 kg/m^2^ (SD: 5.4; range 19.8–44.6).

In two knees, ACL-reconstruction was performed within the same surgery as the implantation of OUKR. 

Of the initial consecutive series (228 knees in 211 patients), three patients had died and 28 patients (29 knees) were lost to follow-up, either due to current residency abroad or refusal to further participate in this trial.

Six patients (six knees) underwent revision surgery. Three of those revisions were performed due to OA progression to the lateral and/or patellofemoral compartment, resulting in the implantation of a TKR at 12, 19 and 27 months after initial operation.

One knee was revised within the first week due to ongoing pain and impaired range of motion (ROM) due to tibiofemoral overstuffing. A further resection of the tibia was performed, and the tibia was exchanged to a cemented version.

There were two cases of inlay dislocation. One appeared at 11 months postoperatively, after the patient stepped out of a car. The second one occurred at three months after surgery without a traumatic cause, when the patient moved the leg while lying down. In this case the tibial component had subsided into a valgus position. Both cases underwent revision surgery with exchange of the inlay. 

One case of arthrofibrosis was reported three months after surgery, with an impaired ROM of 0° – 5° – 80° (Extension–Flexion) in spite of intensive physiotherapy. After closed mobilization under anesthesia, a passive flexion of 120° and an active flexion of >90° at the time of discharge was achieved.

One patient underwent re-operation for suspected infection two weeks after the primary implantation. A debridement with implant retention and joint-lavage was performed. Microbiological examination revealed no bacterial growth. In the postoperative course the patient reported a satisfactory outcome with no signs of further infection. These two cases were seen as re-operations.

**Figure 3:**
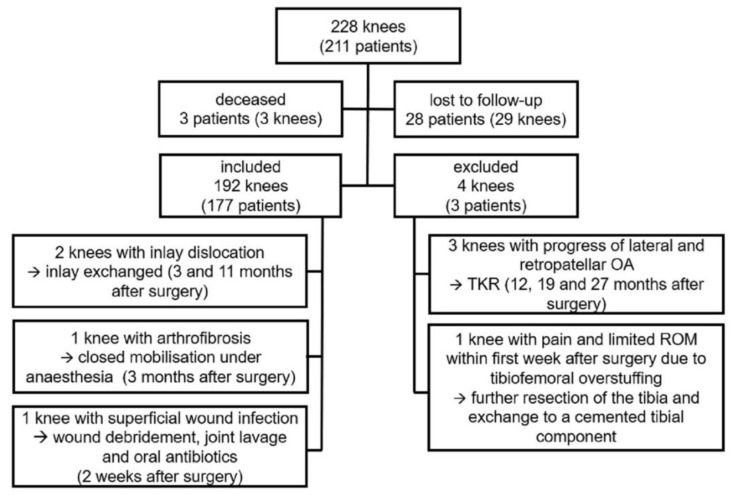
Overview of patient collective.

Figure 3 shows a summary of the patient cohort. 

### 3.1. Survival

Using implant revision as endpoint, the cumulative two year survival was 97.5% (95%-CI: 95.3%−99.7%) and the three year survival 96.9% (95%-CI: 94.4%–99.3%). 

**Figure 4:**
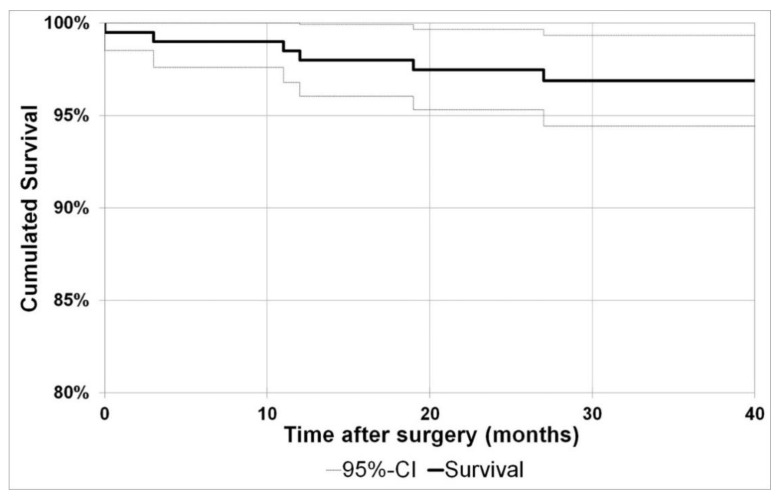
Kaplan–Meier curve: endpoint implant revision .

With re-operation as endpoint, the cumulative two year survival was at 96.5% (95%-CI: 94.0%–99.0%) and the cumulated three year survival at 95.9% (95%-CI: 93.2%–98.6%). Figure 4 shows the Kaplan–Meier curve with the endpoint implant revision.

### 3.2. Clinical Outcome

The clinical outcome scores are presented in Table 1. All score changes from preoperative to follow-up values demonstrated a significant improvement (*p* < 0.001). 

Patients described the outcome of 90.6% of the knees as “satisfactory” or better. The outcome was described by 29.2% as “extremely satisfactory”, 41.7% as “very satisfactory”, 19.8% as “satisfactory”, 7.3% as “sufficient” and 2.1% as “unsatisfactory”.

### 3.3. Radiological Outcome

Radiographs with a minimum follow-up of 24 months after surgery were available for 164 knees (152 patients).

#### 3.3.1. Radiolucent Lines

RL were seen on 36% of the radiographs taken between three months and one year after surgery (96 radiographs available). All of them were partial. Within the first year after surgery, one partial femoral RL was detected in zone 7, which had disappeared three years after surgery. All other RL were seen around the tibial components. Of all detected RLs, 86% had already been present between three and six months after surgery. After a follow-up between one and two years, only 5% of radiographs showed radiolucencies, and the incidence further decreased to 2% at the last follow-up after a minimum of two years. 

RL appeared predominately in tibial zone 6 (34 knees) and zones 5 and 4 (14 each). Five knees showed RL in zone 3, two knees in zone 2 and six knees in zone 1. 

#### 3.3.2. Implant Positioning

The value of tibial and femoral angles, measured as shown in Figure 2, are shown in Table 2 and Table 3. The extent to which these angles deviated from tolerated reference values is presented in Table 4.

Tibial angles within the target range at the last follow-up were observed in 83.3% of the knees with RL at any point, and in 83.6% of those without RL. This difference was not significant. As there was only one femoral RL, no correlation between femoral RL and femoral angles was performed. 

The mean values of tibial and femoral angles did not show any difference between knees with and without RL (*p* > 0.01). Further, the extent to which values exceeded reference values was not significantly different (*p* > 0.01).

One patient, who had suffered an inlay dislocation three months after surgery, showed an increase in tibial slope of 2° and a valgus subsidence of 5° prior to revision surgery. Additionally, one patient showed a valgus subsidence of 5.1° and increase in tibial slope of 1.8° at follow-up in comparison to the postoperative radiograph, but did not report any pain or other clinical problems.

#### 3.3.3. Relation between Radiological and Clinical Outcome

There were no significant differences in the clinical outcomes (OKS, AKSS-O, AKSS-F, FFbH-OA, ROM, pain and satisfaction; *p* > 0.01) between patients with and without RL.

Moreover, for each of the angles, outliers at last follow-up did not have an inferior clinical outcome compared to those within the target ranges (*p* > 0.01).

## 4. Discussion

We evaluated the clinical and radiological outcome of 192 cementless OUKR (177 patients), consecutively implanted between October 2012 and October 2015 at our institution. For clinical evaluation, AKSS, OKS, FFbH-OA, ROM, pain and satisfaction were employed. Fluoroscopically aligned radiographs were analyzed for the incidence of radiolucent lines, as well as implant positioning.

All clinical outcome scores showed excellent results (mean OKS 42.3, SD: 5.9; mean AKSS-O 89.7, SD: 12.8) and significant improvements to their pre-operative equivalents (mean ∆AKSS-O 39.4, SD: 16.7; mean ∆OKS 14.4, SD: 7.7). 

These results are consistent with findings in the literature: van Dorp et al. described OKS results of 43.3 and AKSS of 94.5, with a mean follow-up of 19.5 months [11]. Blaney et al. described a five year follow-up with a mean OKS of 42.0 [10]. 

More than 90% of the patients were at least satisfied with the implant. In the literature, patient satisfaction for UKR above 90% has been reported repeatedly [18,19].

These are better results than for TKR, as displayed in the systematic review of patient satisfaction by Kahlenberg et al. In this review, the majority of reported studies showed more than 80% satisfaction for TKA, while still about one out of five patients might be dissatisfied with the outcome [20].

Concerning the survival analysis, our results of 97.5% two year survival (95%-CI: 95.3%–99.7%) and 96.9% three year survival (95%-CI: 94.4%–99.3%) align with the recently published literature. Campi et al. showed a cumulated five year survival for cementless UKR of 95.8% (mean follow-up 2.7 years). Blaney et al. presented a cumulated five year survival of 98.5%, and the systematic review of van der List et al. reported 96.4% five year survival for cohorts and 96.6% for registries [10,12,21].

In this trial, revision surgery was due to progress of OA and inlay dislocation, aligning with the mentioned reasons in the literature: the systematic review of van der List et al. identified progression of osteoarthritis, bearing dislocation and aseptic loosening as most common causes for failure in cementless UKR. The cohort of Liddle et al. showed progress of osteoarthritis, bearing dislocation, fractures and infection as most common causes for failure [9,12].

There were no cases of tibial plateau fracture in our cohort. This could be attributed to the fact that all surgeons at our institution were specifically trained and experienced with this operation.

In literature, the incidence of radiolucent lines ranges between 7% and 17% [8,22]. However, the time at which the radiograph was taken seems to be of utmost importance. 

Pandit et al. described an incidence of RL at six months of 30% (9/30), which decreased to 6.7% (2/30) at one year [22]. Our cohort showed slightly higher rates, but a similar regression of RL over time. Of the knees with a radiographic follow-up within one year, 36% showed partial RL, 86% of these were already detectable within three to six months after surgery. At follow-up between one and two years, only 5% of the knees showed RLs, and at last follow-up the rate decreased to a mere 2%. This suggests that most RL appear within six months after surgery and regress from then on. As clinical outcome in knees with and without RL were not significantly different, the initially higher incidence of RL in our collective appears to be without any clinical consequences. 

While in TKR, small variations in component alignment may change leg alignments and increase failure rates, mobile bearing UKR seems to be more forgiving with respect to implant positioning. Additionally, mobile-bearing UKR reportedly tolerate alignment deviations better than fixed-bearing UKR [23].

The defined target ranges of the four angles of implant positioning are similar to other cohorts [24,25]. In general, there is a number of studies reporting a notable number of implants outside the recommended ranges with good functional outcome [24,25,26,27].

Gulati et al. examined implant alignment and concluded femoral malalignment of 10° and tibial malalignment of 5° to be tolerable. Within these ranges, no differences in clinical outcome were found, but due to a low rate of outliers, no definitive conclusion as to possible differences in clinical outcome with implant positions outside these ranges was made [23].

We found no significant difference in terms of clinical outcome between patients within and outside the recommended ranges (as shown in Table 4). This aligns with findings for UKR in the literature [28,29].

While exact positioning may be desirable, the tolerated ranges of angles seem to be higher than previously assumed. However, these are only short- to mid-term results, thus studies with longer follow-up periods are needed for clarification.

There was no significant correlation between RL and outliers of implant positioning, which is consistent with the findings by Gulati et al. [23].

Therefore, implant positioning and fixation donot seem to be affected by the presence of RL [30].

Liddle et al. reported on six cementless OUKR which had subsided into valgus and an increased tibial slope, causing pain [31]. We also found two patients with a notable difference in tibial implant positioning angles at follow-up compared to postoperative radiographs, with valgus subsidence and an increase in tibial slope. One of them needed revision surgery due to inlay dislocation. The other one had not reported any noticeable problems. 

The main weakness of this study is the retrospective design, as well as the lack of a control group. Therefore, a multicenter prospective randomized trial, comparing the outcome of cementless and cemented OUKR, is already in the registration process and approved by our institutional review board.

A strength of this study is its larger number of patients. Additionally, the numerous acquired clinical and radiological parameters allow a detailed evaluation of the cementless OUKR. 

## 5. Conclusions

Cementless OUKR shows excellent survival rates after two years and three years of follow-up with good to excellent clinical outcome and high patient satisfaction.

Radiolucent lines appear frequently within the first six months after surgery, and regress over the following time in most cases, suggesting a secure osteointegration of the implant. Their incidence is neither associated with inferior clinical outcome nor with implant positioning. 

Randomized-controlled trials with longer follow-up are needed to confirm the present findings with a higher level of evidence.

## Figures and Tables

**Table 1:** Clinical outcome.

	Mean Last Follow-Up	Mean Pre-op	Mean ∆	*p*-Value
**OKS**	42.3 (SD: 5.9; IQR: 41.0–46.8)	27.9 (SD: 7.2; IQR: 23–33)	14.4 (SD: 7.7; IQR: 9–19.8)	<0.001
**AKSS-O**	89.7 (SD: 12.8; IQR: 86–99)	50.4 (SD: 12.6; IQR: 41–57)	39.4 (SD: 16.7; IQR: 29–50)	<0.001
**AKSS-F**	89.3 (SD: 15.0; IQR: 80–100)	60.7 (SD: 19.8; IQR: 50–70)	28.6 (SD: 20.3; IQR: 10–45)	<0.001
**FFbH-OA**	88.2 (SD: 14.2; IQR: 83–100)	65.1 (SD: 17.0; IQR: 53–78)	23.1 (SD: 17.2; IQR: 11–33)	<0.001
**ROM**	126.1° (SD: 8.4; IQR: 120–130)	122.4° (SD: 13.1; IQR: 120–130)	4.0 (SD: 12.8; IQR: −5–10)	<0.001
**Pain**	1.4 (SD: 2.1; IQR: 0–2)	7.3 (SD: 1.9; IQR: 6–9)	−5.9 (SD: 2.7; IQR: −8–−4)	<0.001

**Table 2:** Tibial implant position angles (values in °). Positive values indicate varus and posterior downslope. Negative values indicate valgus and anterior downslope. n = 164.

	Varus–Valgus			Slope		
	Post-op	Last Follow-Up	Change	Post-op	Last Follow-Up	Change
**Mean**	2.5	2.0	1.1	−2.8	−1.9	1.2
**SD**	2.6	2.7	1.2	2.4	2.6	1.3
**IQR**	1.0–4.2	0.4–3.9	0.3–1.7	−4.5–−1.6	−3.7–−0.3	0.4–1.8
**Outliers**	14 (8.5%)	11 (6.7%)	-	20 (12.2%)	17 (10.4%)	-

**Table 3:** Femoral implant position angles (values in °). Positive values indicate varus and flexion. Negative values indicate valgus and extension. n = 164.

	Varus–Valgus			Flexion		
	Post-op	Last Follow-Up	Change	Post-op	Last Follow-Up	Change
**Mean**	−2.5	−2.3	0.7	10.1	10.2	1.0
**SD**	2.8	2.7	0.7	4.4	4.5	1.0
**IQR**	−4.6–−0.7	−4.0–−0.5	0.1–1.0	7.2–12.7	7.8–12.8	0.3–1.5
**Outliers**	2 (1.2%)	0 (0%)	-	14 (8.5%)	15 (9.1%)	-

**Table 4:** Extent of deviations from recommended ranges for all angles, measured postoperatively and at follow-up.

	Post-op	Follow-Up
**Tibial varus/valgus**	Mean 2.0	Mean 2.4
	SD 1.4	SD 1.4
	IQR 1.0–2.50	IQR 0.9–3.4
**Tibial Slope**	Mean 1.6	Mean 1.4
	SD 1.3	SD 1.1
	IQR 0.6–2.3	IQR 0.6–2.2
**Femoral Varus/Valgus**	Mean 1.5	-
	SD 1.8	
	IQR 0.20–1.45	
**Femoral Flexion/Extension**	Mean 3.9	Mean 3.8
	SD 3.9	SD 3.4
	IQR 1.1–6.3	IQR 1.0–6.0

## References

[1] Green M., Howard P., Porter M., Wilkinson M., Wishart N., National Joint Registry for England Wales Northern Ireland and the Isle of Man National Joint Registry for England, Wales, Northern Ireland and the Isle of Man: 14th Annual Report 2017. http://www.njrreports.org.uk/Portals/0/PDFdownloads/NJR%2014th%20Annual%20Report%202017.pdf.

[2] Liddle A.D., Judge A., Pandit H., Murray D.W. (2014). Adverse outcomes after total and unicompartmental knee replacement in 101,330 matched patients: A study of data from the National Joint Registry for England and Wales. Lancet.

[3] Lombardi A.V., Berend K.R., Walter C.A., Aziz-Jacobo J., Cheney N.A. (2009). Is recovery faster for mobile-bearing unicompartmental than total knee arthroplasty?. Clin. Orthop..

[4] Burn E., Liddle A.D., Hamilton T.W., Pai S., Pandit H.G., Murray D.W., Pinedo-Villanueva R. (2017). Choosing Between Unicompartmental and Total Knee Replacement: What Can Economic Evaluations Tell us? A Systematic Review. Pharm. Open.

[5] The New Zealand Orthopaedic Association The New Zealand Joint Registry―Nineteen Year Report January 1999 to December 2017, 2018. https://nzoa.org.nz/system/files/DH8152_NZJR_2018_Report_v6_4Decv18.pdf.

[6] Campi S., Pandit H., Hooper G., Snell D., Jenkins C., Dodd C.A., Maxwell R., Murray D.W. (2018). Ten-year survival and seven-year functional results of cementless Oxford unicompartmental knee replacement: A prospective consecutive series of our first 1000 cases. Knee.

[7] Gulati A., Chau R., Pandit H.G., Gray H., Price A.J., Dodd C.A., Murray D. (2009). The incidence of physiological radiolucency following Oxford unicompartmental knee replacement and its relationship to outcome. J. Bone Jt. Surg. Br. Vol..

[8] Kerens B., Schotanus M.G., Boonen B., Boog P., Emans P.J., Lacroix H., Kort N.P. (2017). Cementless versus cemented Oxford unicompartmental knee arthroplasty: Early results of a non-designer user group. Knee Surg. Sports Traumatol. Arthrosc..

[9] Liddle A.D., Pandit H., O’brien S., Doran E., Penny I.D., Hooper G.J., Burn P.J., Dodd C.A., Beverland D.E., Maxwell A.R. (2013). Cementless fixation in Oxford unicompartmental knee replacement: A multicentre study of 1000 knees. Bone Jt. J..

[10] Blaney J., Harty H., Doran E., O’Brien S., Hill J., Dobie I., Beverland D. (2017). Five-year clinical and radiological outcomes in 257 consecutive cementless Oxford medial unicompartmental knee arthroplasties. Bone Jt. J..

[11] van Dorp K.B., Breugem S.J., Bruijn D.J., Driessen M.J. (2016). Promising short-term clinical results of the cementless Oxford phase III medial unicondylar knee prosthesis. World J. Orthop..

[12] van der List J.P., Sheng D.L., Kleeblad L.J., Chawla H., Pearle A.D. (2017). Outcomes of cementless unicompartmental and total knee arthroplasty: A systematic review. Knee.

[13] Pandit H., Jenkins C., Gill H.S., Smith G., Price A.J., Dodd C.A., Murray D.W. (2011). Unnecessary contraindications for mobile-bearing unicompartmental knee replacement. J. Bone Jt. Surg. Br. Vol..

[14] Pandit H., Jenkins C., Barker K., Dodd C.A., Murray D.W. (2006). The Oxford medial unicompartmental knee replacement using a minimally-invasive approach. he Journal of bone and joint surgery. J. Bone Jt. Surg. Br. Vol..

[15] Goodfellow J., O’Connor J., Dodd C., Murray D.W. (2011). Unicompartmental Arthroplasty with the Oxford Knee.

[16] Haase I., Schwarz A., Burger A., Kladny B. (2001). Comparison of Hannover Functional Ability Questionnaire (FFbH) and the SF-36 subscale “Physical Functioning”. Die Rehabil..

[17] Zimmer Biomet Oxford Partial Knee―Microplasty Instrumentation―Surgical Technique 2017. https://www.zimmerbiomet.com/content/dam/zimmer-biomet/medical-professionals/000-surgical-techniques/knee/oxford-partial-knee-microplasty-instrumentation-surgical-technique.pdf.

[18] Streit M.R., Streit J., Walker T., Bruckner T., Kretzer J.P., Ewerbeck V., Merle C., Aldinger P.R., Gotterbarm T. (2017). Minimally invasive Oxford medial unicompartmental knee arthroplasty in young patients. Knee Surg. Sports Traumatol. Arthrosc..

[19] Von Keudell A., Sodha S., Collins J., Minas T., Fitz W., Gomoll A.H. (2014). Patient satisfaction after primary total and unicompartmental knee arthroplasty: An age-dependent analysis. Knee.

[20] Kahlenberg C.A., Nwachukwu B.U., McLawhorn A.S., Cross M.B., Cornell C.N., Padgett D.E. (2018). Patient Satisfaction After Total Knee Replacement: A Systematic Review. HSS J..

[21] Campi S., Pandit H.G., Oosthuizen C.R. (2018). The Oxford Medial Unicompartmental Knee Arthroplasty: The South African Experience. J Arthroplast..

[22] Pandit H., Jenkins C., Beard D.J., Gallagher J., Price A.J., Dodd C.A., Goodfellow J.W., Murray D.W. (2009). Cementless Oxford unicompartmental knee replacement shows reduced radiolucency at one year. J. Bone Jt. Surg. Br. Vol..

[23] Gulati A., Chau R., Simpson D.J., Dodd C.A., Gill H.S., Murray D.W. (2009). Influence of component alignment on outcome for unicompartmental knee replacement. Knee.

[24] Walker T., Heinemann P., Bruckner T., Streit M.R., Kinkel S., Gotterbarm T. (2017). The influence of different sets of surgical instrumentation in Oxford UKA on bearing size and component position. Arch. Orthop. Trauma Surg..

[25] Kerens B., Schotanus M.G., Boonen B., Kort N.P. (2015). No radiographic difference between patient-specific guiding and conventional Oxford UKA surgery. Knee Surg. Sports Traumatol. Arthrosc..

[26] Shakespeare D., Ledger M., Kinzel V. (2005). Accuracy of implantation of components in the Oxford knee using the minimally invasive approach. Knee.

[27] Tu Y., Xue H., Ma T., Wen T., Yang T., Zhang H., Cai M. (2017). Superior femoral component alignment can be achieved with Oxford microplasty instrumentation after minimally invasive unicompartmental knee arthroplasty. Knee Surg. Sports Traumatol. Arthrosc..

[28] Clarius M., Hauck C., Seeger J.B., Pritsch M., Merle C., Aldinger P.R. (2010). Correlation of positioning and clinical results in Oxford UKA. Int. Orthop..

[29] Koh I.J., Kim J.H., Jang S.W., Kim M.S., Kim C., In Y. (2016). Are the Oxford((R)) medial unicompartmental knee arthroplasty new instruments reducing the bearing dislocation risk while improving components relationships? A case control study. Orthop. Traumatol. Surg. Res..

[30] Kendrick B.J., James A.R., Pandit H., Gill H.S., Price A.J., Blunn G.W., Murray D.W. (2012). Histology of the bone-cement interface in retrieved Oxford unicompartmental knee replacements. Knee.

[31] Liddle A.D., Pandit H.G., Jenkins C., Lobenhoffer P., Jackson W.F., Dodd C.A., Murray D.W. (2014). Valgus subsidence of the tibial component in cementless Oxford unicompartmental knee replacement. Bone Jt. J..

